# Molecular Dynamics Study on the Reverse Osmosis Using Multilayer Porous Graphene Membranes

**DOI:** 10.3390/nano8100805

**Published:** 2018-10-09

**Authors:** Zhongqiang Zhang, Fujian Zhang, Zhen Liu, Guanggui Cheng, Xiaodong Wang, Jianning Ding

**Affiliations:** 1 Micro/Nano Science and Technology Center, Jiangsu University, Zhenjiang 212013, China; 2211703054@stmail.ujs.edu.cn (F.Z.); ggcheng@ujs.edu.cn (G.C.); wangxd@ujs.edu.cn (X.W.); 2 Jiangsu Collaborative Innovation Center of Photovoltaic Science and Engineering, Changzhou University, Changzhou 213164, China; 3 State Key Laboratory of Structural Analysis for Industrial Equipment, Department of Engineering Mechanics, Faculty of Vehicle Engineering and Mechanics, Dalian University of Technology, Dalian 116024, China; 4 School of Naval Architecture and Ocean Engineering, Jiangsu University of Science and Technology, Zhenjiang 212003, China; liuzhen@just.edu.cn

**Keywords:** porous graphene membranes, reverse osmosis, energy barrier, molecular dynamics

## Abstract

In this study, the reverse osmosis (RO) of a salt solution was investigated using a molecular dynamics method to explore the performance of a multilayer porous graphene membrane. The effects of the salt solution concentration, pressure, layer separation and pore offset on the RO performance of the membrane were investigated and the influences of the number of layers and the gradient structure were determined. The results show that as the salt solution concentration increases, the energy barrier of the water molecules passing through the bilayer porous graphene membranes changes slightly, indicating that the effect of the water flux on the membrane can be ignored. The salt rejection performance of the membrane improves with an increase in the concentration of the salt solution. When the pressure is increased, the energy barrier decreases, the water flux increases and the salt rejection decreases. When the layer separation of the bilayer porous graphene membrane is the same as the equilibrium spacing of the graphene membrane, the energy barrier is the lowest and the membrane water flux is the largest. The energy barrier of the bilayer porous graphene membrane increases with increasing layer separation, resulting in a decrease in the water flux of the membrane. The salt rejection increases with increasing layer separation. The water flux of the membrane decreases as the energy barrier increases with increasing pore offset and the salt rejection increases. The energy barrier effect is more pronounced for a larger number of graphene layers and the water flux of the membrane decreases because it is more difficult for the water molecules to pass through the porous graphene membrane. However, the salt rejection performance improves with the increase in the number of layers. The gradient pore structure enhances the energy barrier effect of the water molecules permeating through the membrane and the water flux of the membrane decreases. The salt rejection performance is improved by the gradient pore structure. The research results provide theoretical guidance for research on the RO performance of porous graphene membranes and the design of porous graphene membranes.

## 1. Introduction

Desalination is a process that removes mineral components from sea water (accounting for 97.5% of global water resources) and has become an important technology to solve the lack of fresh water resources. Desalination has seen important improvements in energy efficiency, reliability, and economics since the 1960s due to numerous advances in reverse osmosis (RO) technology [1]. At present, there are two main methods of seawater desalination: the thermal method and the membrane method [2,3,4,5,6]. The RO method is a type of membrane method and has the advantages of no phase changes, low energy consumption, and high desalination efficiency [7]. It has become the mainstream desalination method and the performance of the membranes used in RO is an important research topic. At present, the commercial RO method has relatively low energy use when a recovery unit and a high-efficiency water pump are used; however, the RO membrane design uses the polyamide membranes composites that were developed 30 years ago. The water flux of the material has only increased about two-fold in the past 20 years and the performance of commercial RO membranes such as cellulose acetate membranes and polyamide membranes has not improved significantly in terms of selectivity and permeability [8,9,10]. The performance of RO membranes and alternative membrane materials such as zeolites, ceramics, and metal-organic frameworks has been investigated [11,12,13]; however, due to manufacturing costs and inherent performance defects, large-scale applications have not been realized. Nanomaterials have been evaluated as RO membranes to improve the salt ion rejection and water flux [14,15].

The excellent physical and chemical properties of single-layer porous graphene represent good application potential of the material for gas screening and RO seawater desalination [16]. Researchers have found that single-layer porous graphene membranes result in high salt ion rejection and a high water flux [17,18]. However, the preparation of single-layer graphene membranes was challenging in the experiment. Although a large number of studies have investigated the continuously improving preparation methods of graphene such as particle bombardment and chemical etching, the cost of preparing large-area single-layer graphene membranes remains very high and the material is prone to cracks and overlap of graphene sheets, which prevents the practical application of single-layer porous graphene membranes in RO seawater desalination [19,20,21,22]. The preparation of multilayer graphene membranes is simpler than that of single-layer graphene sheets membranes and the efficiency is higher and the cost is lower. A multilayer graphene membrane is more hydrophobic than a single-layer, which is advantageous for trapping salt ions and increasing the salt ion rejection [23]. Therefore, it is of great significance to investigate the performance of multilayer graphene membranes for RO. Few studies have investigated this topic and the effects of the number of layers of the graphene membrane and the structural changes in the pores caused by the number of layers on the RO performance are unclear.

Regarding the molecular simulation of graphene, the researchers have studied the properties and application direction of graphene by Ab-initio molecular dynamics, which is of great significance for the further development of graphene [24,25]. Molecular dynamics (MD) simulations have also been successfully paired with ab-initio molecular dynamics, in order to model the behavior of systems in which classical potentials cannot properly account for the physics at play. This approach has yielded insights into the dynamics of water passing through graphene membrane as well as the potential of carbon nanotubes for desalination [26].

In this study, the RO of a salt solution is simulated using the MD method to determine the performance of multilayer porous graphene membranes. The RO performance is determined for different salt solution concentrations, driving zone pressure, pore offsets, layer separations and number of layers. The results of this study provide an improved understanding of RO of a salt solution using multilayer graphene membranes and theoretical guidance for the preparation of a RO membrane using graphene.

## 2. Model and Methods

**Figure 1:**
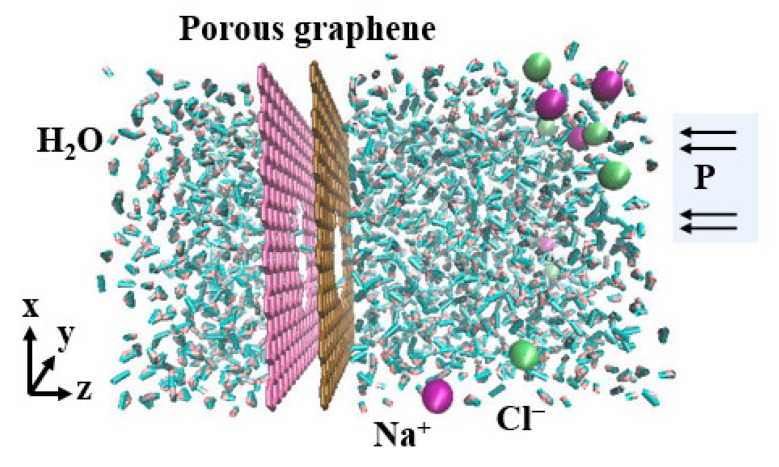
Reverse osmosis model for a salt solution driven by external pressure P. The blue balls are sodium ions, the purple balls are chloride ions, the pink and yellow-brown areas represent the porous graphene membrane and the others features are water molecules.

The initial model of the RO using a bilayer porous graphene membrane is shown in Figure 1. The right driving zone is the salt solution composed of sodium ions, chloride ions and water molecules and the left side is the permeation zone of pure water molecules. The bilayer perforated graphene is shown in the middle of the model. The salt solution has a concentration of 35.1 g/L and there are 1138 water molecules, 16 sodium ions and 16 chloride ions; the left permeation zone is pure water and contains 555 water molecules. In the simulation, the graphene is porous in the directions of the *x*-axes and *y*-axes, the *z*-axis direction is a fixed boundary. The size of the simulation system is 35 Å × 35 Å × 100 Å. The driving region has a higher internal pressure than the osmotic pressure of the salt solution, which is achieved by applying pressure to the graphene plate on the left side of the model so that the salt solution is forced through the porous graphene to separate the water molecules from the salt ions. During the RO process, the salt is entrapped in the driving zone and the water molecules pass through the multilayer porous graphene into the permeation zone.

The interaction between the salt ions, water molecules and carbon atoms in the graphene during the MD simulation is described by the Lennard-Jones (LJ) potential function. The parameters of the LJ potential energy function of the salt ions and water molecules are shown in Table 1 and Table 2 [27,28,29,30]. The interaction between the parameters is determined by the Lorentz-Berthelot mixing criterion and the interaction between the carbon atoms in the graphene is described by the AIREBO potential function [31]. The truncation radii of the LJ interaction and the Coulomb interaction are 10 Å and 12 Å, respectively and the long-range electrostatic interaction is calculated by the particle-particle particle-mesh algorithm [32]. In the RO process, the time step is set to 1 fs and the number of relaxation steps is 50,000. After the system has stabilized, the RO process is simulated; the time step is 3,000,000 and the temperature of the system is controlled by a thermostat algorithm at 300 K.

## 3. Results and Discussion

**Figure 2:**
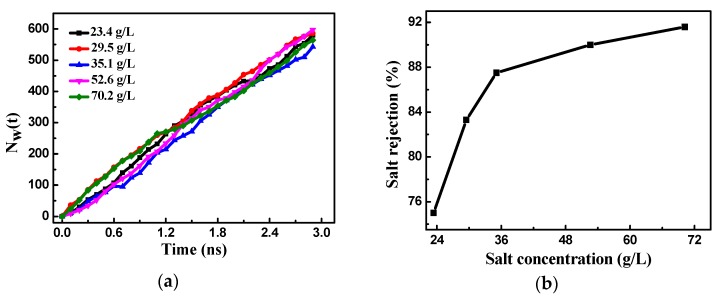
(**a**) The number of water molecules passing through the bilayer porous graphene membranes over time for different salt concentrations; (**b**) effect of salt concentration on the salt rejection of the bilayer porous graphene membranes.

The concentration of the salt solution affects the RO and even changes the structure and resulting performance of the membrane, affecting its service life. Therefore, it is necessary to consider the effect of the salt solution concentration on the bilayer porous graphene membranes. The concentration of the salt solution in the simulation ranged from a salinity lower than seawater (23.4 g/L) to higher than seawater (70.2 g/L). Figure 2a shows the number of water molecules passing through the bilayer porous graphene membranes over time for different salt concentrations. The results indicate that the number of water molecules increases approximately linearly over time and the concentration of the salt solution has little effect on the water flux of the bilayer porous graphene membranes.

Figure 2b shows the effect of the salt concentration on the salt rejection of the bilayer porous graphene membranes. The salt rejection increases with an increase in the salt solution concentration. When the salinity is lower than the seawater salinity, the salt rejection increases rapidly with the increase in the concentration of the salt solution. In contrast, the salt rejection increases at a lower rate when the salinity is higher than the seawater salinity. The effect of the salt solution concentration on the RO performance is similar for the bilayer and multilayer porous graphene membranes. The salt rejection performance of the bilayer porous graphene membranes increases with the increase in the salt solution concentration; however, the effect on the water flux of the membrane is relatively small. In order to optimize the performance of the RO membrane, the salt solution concentration in the subsequent simulation is higher than the seawater salinity.

In order to obtain a better understanding of the effects of the salt solution, the change in free energy of the water molecules passing through the bilayer porous graphene membrane is determined. The highest free energy value in the free energy potential energy curve in the *z*-axis direction represents the energy barrier when the water molecules pass through the membrane. The higher the energy barrier of the membrane structure, the more energy is required for the water molecules to pass through the bilayer porous graphene membranes, the more difficult it is for the water molecules to pass through, and the smaller the water flux of the membrane is and vice versa.

**Figure 3:**
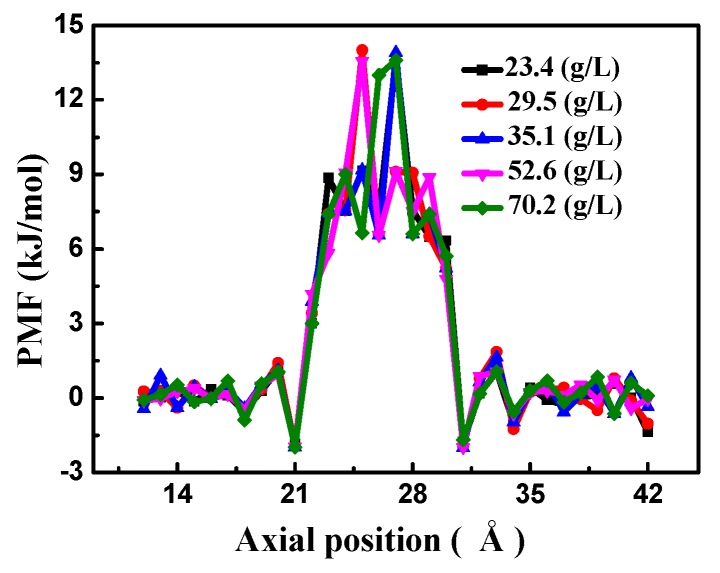
The potential of mean force (PMF) of the bilayer porous graphene membrane as a function of the salt concentration.

We used the Boltzmann statistics method to determine the potential of mean force (PMF): *F(r) = −RTln*[*ρ(r)*]. Where *R* is the gas constant, *T* is the temperature, and *ρ(r)* is the density of the salt solution at the *r* position. Common calculation methods for the free energy are the thermodynamic integration method and the sampling method. It is simpler and more efficient to use the Boltzmann sampling method than the thermodynamic integration method. As shown in Figure 3, the free energy has a peak at 22–30 Å in the *z*-axis direction, which is the energy barrier of the water molecules passing through the bilayer porous graphene membrane. The energy barrier is similar for the different salt solution concentrations, indicating that the salt concentration has little effect on the water molecules passing through the membrane. This result is in agreement with the effect of the salt solution on the number of water molecules passing through the membrane and on the water flux, indicating that the salt solution can be ignored in the simulation.

**Figure 4:**
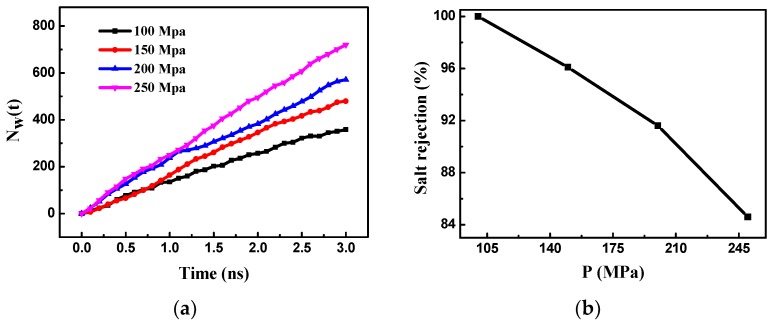
(**a**) The number of water molecules passing through the bilayer porous graphene membrane over time for different external pressures; (**b**) effect of external pressure on the salt rejection of the bilayer porous graphene membrane.

The pressure affects the RO performance and the service life of the RO membrane; therefore, it is necessary to explore the effect of the pressure on the performance of the bilayer and multilayer graphene membranes. The pressure in the driving zone was changed to 100 MPa, 150 MPa, 200 MPa, and 250 MPa and the relationship between the number of permeating water molecules and the pressure was obtained (Figure 4a). The number of permeating water molecules increases almost linearly over time, indicating that the number of water molecules passing through the membrane is constant in unit time. The greater the pressure, the more water molecules pass through the bilayer porous graphene membrane. As the pressure increases, the water flux of the membrane increases. At a pressure of 250 MPa, the number of water molecules passing through the membrane and the water flux are highest.

The relationship between the salt rejection of the bilayer porous graphene membrane and the pressure is shown in Figure 4b. As the pressure increases, the salt rejection rate decreases and the salt rejection performance of the bilayer porous graphene membrane deteriorates. The data show that the salt ion rejection rate is highest and the salt ions are completely trapped in the membrane when the pressure is 100 MPa; however, as the pressure increases, the salt ion rejection rate decreases and the effect is the same for the single-layer graphene membrane and the experimental RO membrane, and the membrane water flux and salt ion rejection performance have a mutually constrained phenomenon that one decreases with the increase of the other.

**Figure 5:**
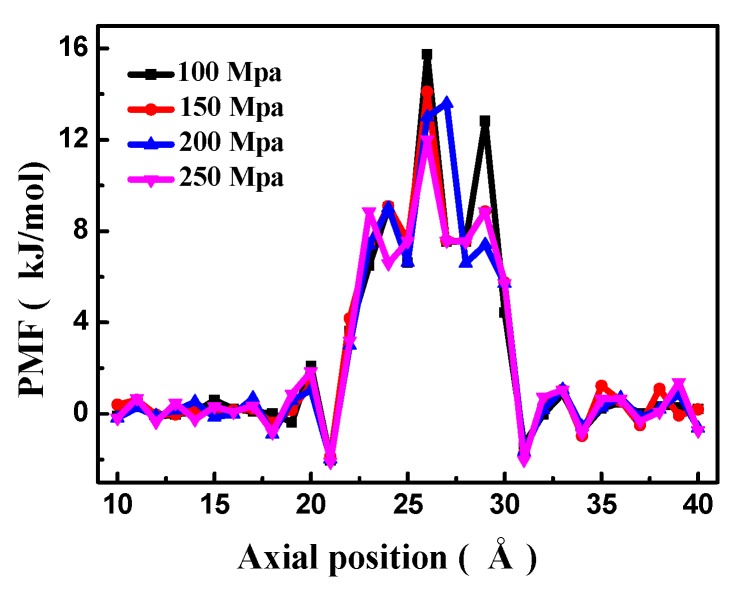
The PMF of the bilayer porous graphene membrane as a function of external pressure.

The effect of different pressures on the PMF of the bilayer porous graphene membrane is shown in Figure 5. The high-energy peak of the free energy shows that the energy barrier of the water molecules passing through the bilayer porous graphene decreases with increasing pressure. The peak of the free energy is highest at a pressure of 100 MPa. When the pressure is increased, the peak value of the free energy decreases. The peak value of the free energy is the lowest at a pressure of 250 MPa. Therefore, when the pressure in the driving zone is 100 MPa, the water molecules have a greater difficulty to pass through the porous graphene membranes than at a pressure of 250 MPa. This result is in agreement with the results shown in Figure 4a.

**Figure 6:**
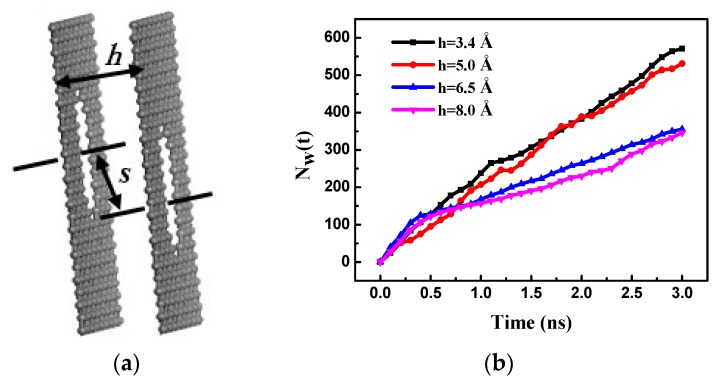
(**a**) Schematic diagram of the bilayer porous graphene membrane with a layer separation *h* and pore offset *s*; (**b**) the number of water molecules passing through the bilayer porous graphene membranes over time for different layer separation distances.

The different layer separation distances of the bilayer porous graphene membrane *h* are 3.4 Å, 5.0 Å, 6.5 Å, and 8 Å; these values are lower than the cutoff radius. The effect of the pore offset on the membrane performance is investigated by changing the offset *s* of the graphene hole centers; the different offset values *s* are 0.0 Å, 2.5 Å, 5.0 Å, and 7.5 Å. A diagram of the layer separation and pore offset of the bilayer graphene membrane is shown in Figure 6a. The salt solution concentration (70.2 g/L) and the driving zone pressure are constant at 200 MPa during the simulated RO process. The number of water molecules passing through the bilayer porous graphene membrane over time for different layer separation distances is shown in Figure 6b. The number of water molecules passing through the membrane increases almost linearly over time. When the layer separation *h* is 3.4 Å (the equilibrium spacing) and 5.0 Å, the number of water molecules passing through the membrane is larger at the same time. When the layer separation *h* increases to 6.5 Å and 8.0 Å, the number of water molecules passing through the membrane is relatively small. Therefore, it is evident that the number of water molecules passing through the membrane decreases with the increase in the layer separation because the water flux of the membrane is reduced, which reduces the RO performance of the porous graphene membrane. It is observed that there is no salt solution between the two layers of the porous graphene membrane when the layer separation *h* is 3.4 Å (the equilibrium spacing) and 5.0 Å whereas the salt solution is present between the layers when the layer separation *h* is 6.5 Å and 8.0 Å. The RO process of the bilayer porous graphene membrane may be similar to the fact that the salt solution twice passes through the single-layer porous graphene membrane.

**Figure 7:**
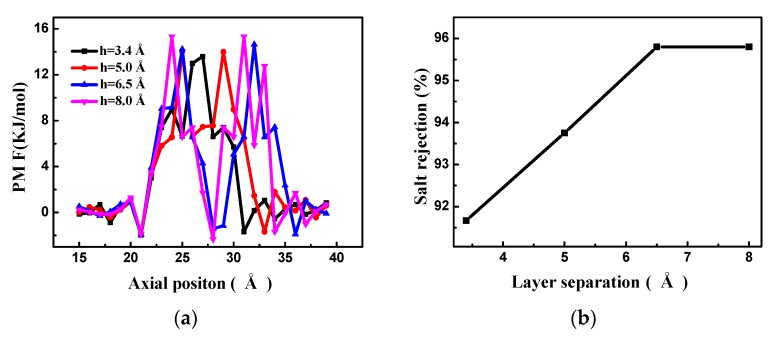
(**a**) The PMF of the bilayer porous graphene membrane for different layer separation distances; (**b**) salt rejection of the bilayer porous graphene membrane as a function of the layer separation.

The PMF of the water molecules passing through the bilayer porous graphene membrane was determined for the different layer separation distances, as shown in Figure 7a. When the layer separation is the same as the equilibrium spacing, only one peak is observed for the PMF, indicating that the lowest energy barrier occurred at the equilibrium spacing. As the layer separation increases, the PMF exhibits two peaks, indicating that the energy barrier is affected by the two layer porous graphene membrane. With further increase in the layer separation, the energy barrier increases, resulting in a decrease in the number of water molecules passing through the bilayer porous graphene membrane, i.e., the water flux of the membrane decreases.

Figure 7b shows the salt rejection of the bilayer porous graphene membrane as a function of the layer separation. When the layer separation is the same as the graphene equilibrium spacing, the salt rejection performance is the lowest. The salt rejection increases with the increase in the layer separation. However, as this occurs, a certain amount of the salt solution is retained between the graphene layers, increasing the concentration polarization between the graphene layers, accelerating the precipitation of the porous graphene membrane, and reducing the RO performance of the porous graphene. A comprehensive analysis of the membrane water flux and salt ion rejection through the different separation layers shows that the increase in the layer separation is not conducive to improving the RO performance of the porous graphene membranes and the cost of preparation increases. Therefore, the graphene equilibrium spacing was selected in the subsequent simulation of the multilayer porous graphene membrane RO process.

**Figure 8:**
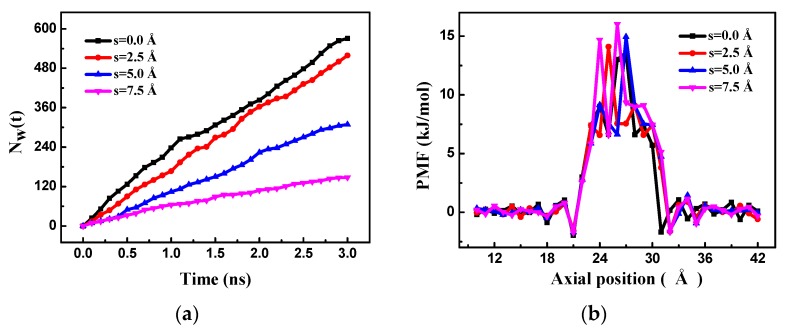
(**a**) The number of water molecules passing through the bilayer porous graphene membrane over time for different pore offsets; (**b**) the PMF of the bilayer graphene membranes for different pore offsets.

The pore offset has an important influence on the RO performance of the bilayer porous graphene membrane. The number of water molecules passing through the bilayer porous graphene membrane over time for different pore offsets is shown in Figure 8a. It is observed that the number of water molecules passing through the membrane increases almost linearly over time for the different pore offsets. When the positions of the two holes overlap completely (*s* = 0 Å), the largest number of water molecules pass through the membrane and the water flux of the membrane is at a maximum. As the pore offset increases, the number of water molecules decreases and when the pore offset is 7.5 Å, the smallest number of molecules passes through the membrane and the water flux of the membrane is at a minimum. Since the layer separation is the same as the equilibrium spacing, the salt solution cannot flow into the graphene sheet; therefore, the position of the two holes cannot be completely staggered and the pore offset does not need to continue increasing. The flow of the water molecules is investigated by counting the water molecules passing through the bilayer porous graphene membrane for the different pore offsets, as shown in Figure 8b. The energy barrier of the water molecules passing through the bilayer porous graphene membrane for the different pore offsets at the equilibrium spacing is the result of the interaction of the bilayer graphene membrane. Therefore, the PMF has only one peak, and the peak value is the smallest, indicating that the smallest energy barrier occurs for the overlapping pore position and the water molecules pass through easily. When the pore offset is 7.5 Å, the peak is the highest, the energy barrier is the highest, and the water molecules cannot penetrate easily. The number of water molecules passing through the membrane decreases as the pore offset increases.

**Figure 9:**
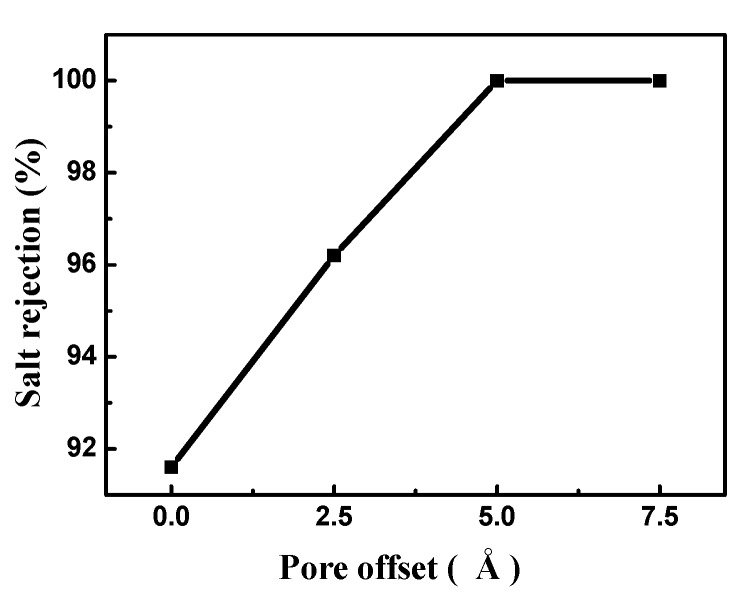
Salt rejection of the bilayer porous graphene membrane for different pore offsets.

The salt rejection of the bilayer porous graphene membrane for different pore offsets is shown in Figure 9. When the pore offset is zero, the salt rejection is low and the salt rejection increases with increasing pore offset. When the pore offset exceeds 5 Å, a complete interception of the salt ions is observed. This occurs because the pore size of the two-hole overlap area is smaller than the hydration radius of the salt ions at the pore offset position and the salt cannot pass through the bilayer porous graphene membrane. The RO performance at this position is similar to that of the single-layer porous graphene membrane when the pore diameter is smaller than the salt hydration radius.

**Figure 10:**
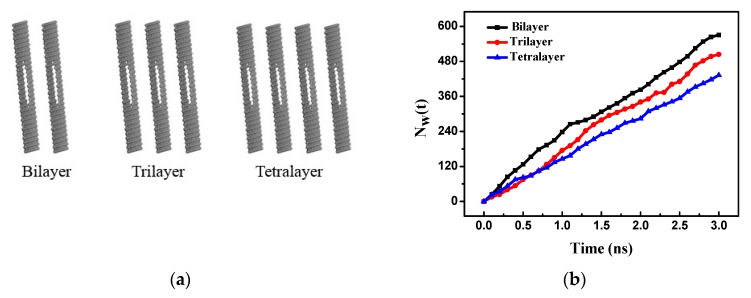
(**a**) Multilayer porous graphenes: bilayer, trilayer and tetralayer; (**b**) the number of water molecules passing through multilayer porous graphene membranes versus time.

It was reported that the hydrophobicity of graphene decreases as the number of layers of graphene increases [33]. Here, the influence of bilayer, trilayer, and tetralayer graphene membranes on the RO performance was investigated, as shown in Figure 10a. Figure 10b shows the number of water molecules passing through the bilayer, trilayer, and tetralayer porous graphene membranes over time. The number of water molecules increases almost linearly over time for the three types of membranes. The largest number of water molecules pass through the bilayer membrane and the water flux is the highest. As the layers increase, the number of water molecules passing through them decreases and the fewest number of water molecules pass through the tetralayer membrane and the water flux of this membrane is the lowest. The water flux of the membrane decreases with the increase in the number of layers.

**Figure 11:**
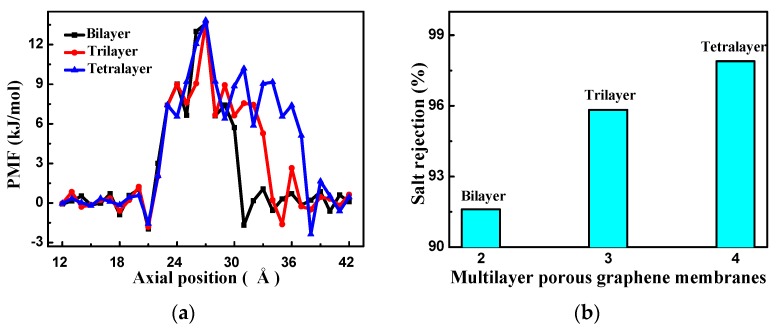
(**a**) The PMF of the multilayer porous graphene membranes; (**b**) salt rejection of the multilayer porous graphene membranes.

Figure 11a shows the PMF of the water molecules passing through the three different membranes. The PMF of the different membranes has only one peak because the layer separation is the same as the equilibrium spacing and the energy barrier for the multilayer membranes is obtained by every layer membrane affecting together. The peaks are similar for the three different membranes; the peak section is shortest for the bilayer and longest for the tetralayer. When the water molecules pass through the tetralayer membrane, the energy barrier effect is more pronounced, indicating that more energy is required to pass through this membrane. With an increasing number of layers, the number of water molecules passing through the membrane and the water flux decrease.

The salt rejection of the different layers of porous graphene membranes is shown in Figure 11b. The salt rejection is lowest for the bilayer membrane and increases with the number of layers. Figure 10b and Figure 11b indicate that the water flux of the multilayer membranes decreases and the salt rejection increases as the number of layers increases. The membrane water flux and salt ion rejection performance still have a mutually constrained phenomenon as the number of layers changes; therefore, the pore structure of the membrane is changed to the gradient pore structure in order to seek high rejection and high water flux RO performance.

**Figure 12:**
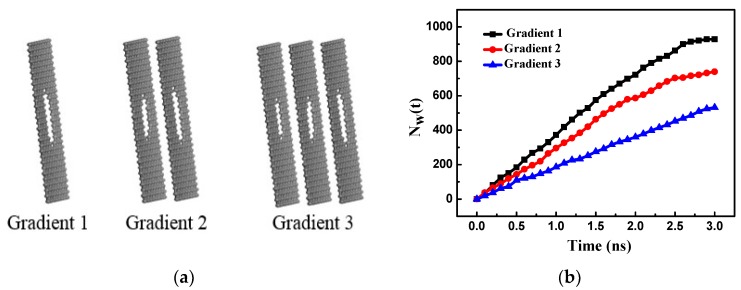
(**a**) Gradient 1, Gradient 2 and Gradient 3 porous graphene membranes; (**b**) the number of water molecules passing through Gradient 1, Gradient 2 and Gradient 3 porous graphene membranes versus time.

**Figure 13:**
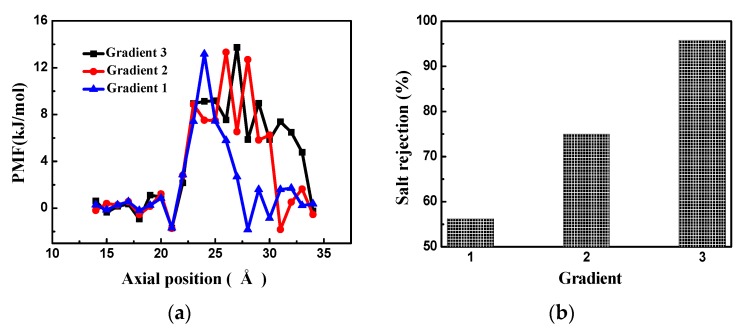
(**a**) The PMF of the porous graphene membranes for Gradient 1, Gradient 2 and Gradient 3; (**b**) salt rejection of the porous graphene membranes for Gradient 1, Gradient 2 and Gradient 3.

The pore gradients of the bilayer and trilayer porous graphene membranes remain unchanged and the RO performance of the single-layer porous graphene membrane is analyzed. As shown in Figure 12a, the first layer of the trilayer porous graphene membrane (Gradient 1) has 13 graphene rings, the second layer (Gradient 2) has 10 graphene rings, and the third layer (Gradient 3) has seven graphene rings. The bilayer membrane consists of the first two layers of the three-layer graphene membrane and the single-layer porous graphene membrane has 13 graphene rings (similar to the first layer graphene membrane in the trilayer porous graphene membrane). This arrangement is used to investigate the effect of the gradient pore structure on the RO performance of the porous graphene membrane. Figure 12b shows the number of water molecules passing through the membranes with the three types of gradient pore structure. The results indicate that the number of water molecules passing through the membrane increases almost linearly over time, the largest number of water molecules pass through the Gradient 1 membrane, and the water flux is highest for Gradient 1. From Gradient 1 to Gradient 3, the number of water molecules passing through the membrane increases slowly and the water flux of the membrane decreases. The flow of the water molecules in the gradient pore is determined by the energy barrier of the water molecules; therefore, the PMF is determined for the three types of gradient pore structures, as shown in Figure 13a. The PMF exhibits only one peak section for each of the three types of gradients. Gradient 3 has the longest peak section because the water molecules require more energy to pass through the membrane; as a result, the smallest water flux is observed for Gradient 3.

The salt rejection is relatively low for Gradient 1 and is highest for Gradient 3 (Figure 13b). From Gradient 1 to Gradient 3, the salt rejection increases and the water flux of the membrane decreases because the gradient pore structure plays the role of layer by layer rejection.

## 4. Conclusions

The RO of a salt solution in a multilayer porous graphene membrane was investigated using a MD method. The effects of the salt solution concentration, pressure, layer separation and pore offset on the RO performance of the membrane were investigated and the influence of the number of layers and the gradient structure were determined.

The results show that as the salt solution concentration increases, the energy barrier of the water molecules passing through the bilayer porous graphene membrane changes only slightly, indicating that the effect of the water flux on the membrane can be ignored. The salt rejection of the membrane increases with the concentration of the salt solution. When the pressure is increased, the energy barrier decreases, the water flux increases, and the salt rejection decreases. When the layer separation of the bilayer porous graphene membrane is the same as the equilibrium spacing of the graphene membrane, the energy barrier is the lowest and the membrane water flux is the largest. The energy barrier of the bilayer porous graphene membranes increases with increasing layer separation, resulting in a decrease in the water flux of the membrane. The salt rejection increases with increasing layer separation. The water flux of the membrane decreases as the energy barrier of the bilayer porous graphene membranes increases with increasing pore offset but the salt rejection increases. When the pore offset is increased, the water flux of the membrane decreases as the energy barrier of the bilayer porous graphene membranes increases but the salt rejection increases.

When the number of graphene layers is increased, the energy barrier effect is more pronounced, and the water flux of the membrane decreases but the salt rejection increases. The gradient pore structure enhances the energy barrier effect and the water flux of the membrane decreases. Nevertheless, the salt rejection performance is improved by the gradient pore structure. The research results provide theoretical guidance for research on the RO performance of porous graphene membranes and the design of porous graphene membranes.

## Figures and Tables

**Table 1:** Parameter *σ* in the Lennard-Jones potential.

*σ* (Å)	C	Na	Cl	H	O
**C**	3.40	2.88	3.90	0.00	3.28
**Na**	-	2.35	3.38	0.00	2.76
**Cl**	-	-	4.40	0.00	3.78
**H**	-	-	-	0.00	0.00
**O**	-	-	-	-	3.16

**Table 2:** Parameter *ɛ* in the Lennard-Jones potential.

*ɛ* (kcal/mol)	C	Na	Cl	H	O
**C**	0.0860	0.1058	0.0929	0.0000	0.1183
**Na**	-	0.1303	0.1144	0.0000	0.1455
**Cl**	-	-	0.1003	0.0000	0.1278
**H**	-	-	-	0.0000	0.0000
**O**	-	-	-	-	0.1628
